# The Role of Platelets in the Tumor-Microenvironment and the Drug Resistance of Cancer Cells

**DOI:** 10.3390/cancers11020240

**Published:** 2019-02-19

**Authors:** Phung Thanh Huong, Lap Thi Nguyen, Xuan-Bac Nguyen, Sang Kook Lee, Duc-Hiep Bach

**Affiliations:** 1 Department of Biochemistry, Hanoi University of Pharmacy, Hanoi 10000, Vietnam; huongpt@hup.edu.vn (P.T.H.); lapnt29@gmail.com (L.T.N.); bac.biochemistry.hup@gmail.com (X.-B.N.); 2 College of Pharmacy, Natural Products Research Institute, Seoul National University, Seoul 08826, Korea

**Keywords:** platelet, drug resistance, platelet-derived growth factor, angiogenesis, metastasis, cancer biomarker

## Abstract

Besides the critical functions in hemostasis, thrombosis and the wounding process, platelets have been increasingly identified as active players in various processes in tumorigenesis, including angiogenesis and metastasis. Once activated, platelets can release bioactive contents such as lipids, microRNAs, and growth factors into the bloodstream, subsequently enhancing the platelet–cancer interaction and stimulating cancer metastasis and angiogenesis. The mechanisms of treatment failure of chemotherapeutic drugs have been investigated to be associated with platelets. Therefore, understanding how platelets contribute to the tumor microenvironment may potentially identify strategies to suppress cancer angiogenesis, metastasis, and drug resistance. Herein, we present a review of recent investigations on the role of platelets in the tumor-microenvironment including angiogenesis, and metastasis, as well as targeting platelets for cancer treatment, especially in drug resistance.

## 1. Introduction 

Despite emerging significant advances in therapeutic strategies, chemotherapy is still considered as a cornerstone in the treatment of various cancer types. Treatment failure is still a tough problem which mostly originates from the drug resistance of cancer cells [1,2,3,4]. Clinically, cancer patients are susceptible to chemotherapy, but over time most of their tumors sooner or later can become resistant after repeated treatment, leading to tumor relapse, metastasis, and limited overall survival [2,5,6,7].

So far, various mechanisms of drug resistance in cancer treatment have been unraveled, eliciting its heterogeneous and multifactorial nature [8]. Platelets have been investigated for decades for their critical role in tumorigenesis. Recently, a number of studies have focused on the association between platelets and drug resistance. Platelets are not only a beneficial ally of tumors in progression and migration but can also stimulate tumors to be resistant to chemotherapy [9,10]. Therefore, understanding of the functional contributions of platelets in cancer drug resistance as well as in the tumor-microenvironment, such as angiogenesis and metastasis, might possibly provide better strategies in cancer treatment. Herein, we highlight the association of platelets–cancer cell interactions in tumor progression as well as in drug resistance. 

## 2. Landscape of Platelets in Cancer—From Bench to Bed 

The critical role of platelets in tumor progression has been investigated for more than 50 years. To date, many different mechanisms of the interactions between cancer cells and platelets have been revealed, most of which involve the recruitment of activated platelets for facilitating tumor growth, angiogenesis, and metastasis. Based on the role of platelets in malignancy pathology, there is an emerging trend for researchers to exploit platelets in cancer diagnosis, prognosis, and treatment.

Originally, the active roles of platelets in promoting cancer growth and invasion led to the idea that an abnormally increased number of platelets might be potential biomarkers for cancer risk. Various cohort studies have reported that cancer incidence increases with increasing platelet count and those with a count of >3.5 × 10^11^/L have more than a 3% risk of cancer in one year of observation [11,12]. This may be a notable observation for primary care in order to attenuate cancer development.

In response to tumor activation signals, there is a dramatic change in the expression of certain platelet-derived proteins [13,14]. Additionally, platelets can sequester and deliver tumor-associated factors for angiogenesis and metastasis. As a result, the platelet proteome of cancer patients is different from that of a normal healthy person (or non-cancer patients). Recent studies have detected platelet protein biomarker candidates that can be applied for early diagnosis of various cancer types [15,16,17]. In addition to diagnosis, platelet quantity is also used for cancer prognosis and treatment monitoring. In pancreatic cancer patients with synchronous liver metastases, the overall survival of those with a mean platelet volume (MPV) of  >8.7 femtoliters (fL) is significantly shorter than that of those with an MPV of ≤8.7 fL [18]. A meta-analysis suggests that a platelet count will be applicable as a prognostic marker in pancreatic cancer [19]. On the contrary, the overall survival of non-small cell lung cancer (NSCLC) patients with a platelet distribution width (PDW) of  ≥16.3% is significantly longer than that of those with a PDW of <16.3% [20]. 

Another promising application of platelets in cancer treatment involves the strong interaction between activated platelets and cancer cells via receptors of high affinity, which can be utilized to design platelet-based drug delivery systems specifically targeting cancer cells, especially metastasizing ones and hematologic malignancies [21,22]. 

At the moment, a targeted tumor promoter or tumor suppressor is elicited to be in the era of personalized medicine. Many biomarkers have been developed in order to ensure both the efficacy and safety of treatment regimens. Among these biomarkers, platelets and their derivatives can potentially be used for the prediction of treatment response and minimization of drug resistance. 

## 3. Platelets in the Tumor-Microenvironment

The tumor-microenvironment consists of not only cancer cells but also a system of different cell types from epithelial and mesenchymal cells to various blood vessel cells and immune cells that play important roles in tumor growth [23,24]. So far, there is more and more evidence of complicated interactions between platelets and various cells of the tumor-microenvironment, in most of which platelets are trained and recruited as favorable supporters for tumor angiogenesis and metastasis. Further, tumor infiltration even makes platelets a part of the tumor-microenvironment. 

### 3.1. Platelets and Tumor Angiogenesis

**Figure 1:**
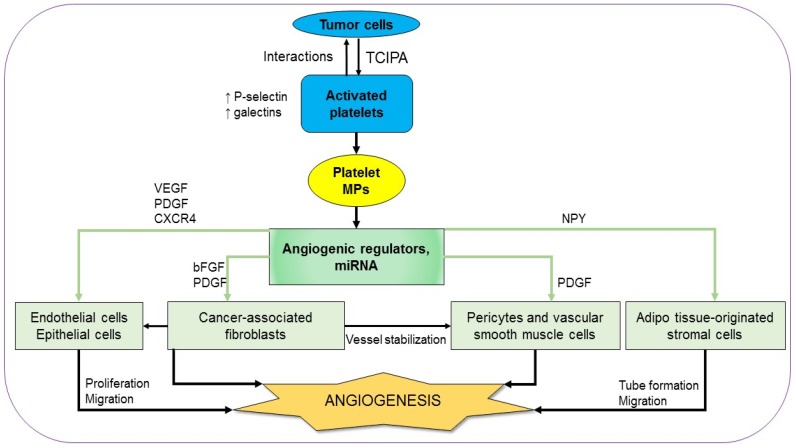
Platelets and tumor angiogenesis. Interactions between tumor cells and activated platelets result in angiogenic regulators and microRNAs which are delivered by platelets microparticles (MPs) to various cell types of the tumor-microenvironment in favor of neovascularization (see text for details).

Angiogenesis is a fundamental process in tumor malignancy and progression which is essential for both solid and non-solid tumors. The active role of platelets in angiogenesis from the early steps of vasculogenesis until the advanced stages has been known for a long time [25,26]. In order to recruit platelets as an ally, tumors firstly activate platelets using their tissue factors (TFs)-containing microparticles (MPs) [27]. The tumor cell-induced platelet activation (TCIPA) is characterized by platelet aggregation, adhesion, and an increase of both platelet quantity and platelet-derived pro-angiogenic factors [28]. The platelets are activated through their essential pathways, including thromboxane (TX)-A2, glycoprotein (GP)-Ib-IX, adenosine diphosphate (ADP), and GPIIb/IIIa [29]. There is a dramatic increase in both platelet activation markers, such as P-selectin, an adherent molecule, and angiogenesis markers in the platelets of various types of cancer patients. For example, platelet lysate from breast cancer patients contains significantly higher levels of vascular endothelial growth factor (VEGF), angiopoietin-1, and P-selectin, compared to that from normal controls [30]. Similarly, intra-platelet levels of VEGF and platelet-derived growth factor (PDGF) augment in colorectal cancer patients compared to those in controls [31]. Notably, these angiogenic regulators are detected at the early phase and their levels are associated with clinical characteristics (Figure 1) [13,30,31]. Using immunohistochemical staining with human colorectal tumor specimens, Qi et al. showed that activated platelets are chemoattractant toward the cancer cells. As soon as the platelets adhere to cancer cells, there is an acceleration of platelet-derived angiogenic regulators to facilitate angiogenesis in the tumor. Both VGEF expression and the impacts of platelets on tumor vascularity are depleted in genetic P-selectin-deficient mice, suggesting that the interactions between activated platelets and cancer cells are mediated by P-selectin [32]. Additionally, the interactions between activated platelets and cancer cells are also mediated by galectins, another kind of cell adhesion molecule. Different members of the galectin family can discriminatingly regulate the release of angiogenic regulators by human platelets [33]. The importance of TCIPA in tumor angiogenesis is supported by the fact that several antiplatelet and anticoagulant agents can inhibit the expression of platelet angiogenic proteins and platelet-induced angiogenic response as well (Figure 1) [28,29,34]. The treatment of thrombin-stimulated human platelets with various inhibitors can also reveal the contribution of multiple signaling pathways in the platelet pro-angiogenic responses. Inhibition of PKC, p38, ERK1/2, Src kinases, or PI3K/Akt pathways partially interferes with endothelial cells growth and tube formation at various levels. For instance, angiogenetic effects were partially but remarkably inhibited by the inhibition of PKC, p38, and ERK1/2 pathways while the blockade of Src kinases or PI3K/Akt showed little or no effect, respectively. However, the platelet-induced endothelial cells growth and tube formation are totally inhibited in the presence of aspirin. Treatment with indomethacin, another COX inhibitor, like aspirin, results in similar impacts, without significantly inhibiting the phosphorylation of p38, ERK, Src, Akt, and PKC substrates [33]. Taken together, it can be said that the overall pro-angiogenic effects of platelet-derived angiogenic regulators are regulated by different signaling pathways but essentially depend on the action of COX. The supportive impacts of platelets in angiogenesis are not only on functional epithelial cells but also on their precursors, the epithelial progenitor cells (EPCs). A co-culture of platelet-rich plasma and EPCs results in the build-up of vasculogenesis-related factors such as VGEF, PDGF, stromal cell-derived factor 1 (SDF-1), and fetal liver kinase 1 (Flk-1), which is followed by the spreading of vessel-like structure formation [35]. Among the platelet-originated angiogenic factors, the growth factors (GFs) play an important role in endothelial cell proliferation and migration. Even a concentrated GF preparation containing PDGF, VEGF, and CXC chemokine receptors 4 (CXCR4) can promote endothelial cell proliferation and migration in a dose-dependent manner in an in vitro study with human umbilical vein endothelial cells (HUVECs). Additionally, the preparation enhances the expression of PDGF, CXCR4, and VEGF in dental pulp cells, which are also known as supports for epithelial cell proliferation (Figure 1) [36,37]. In response to the rise of platelet-derived growth factors, there is a boost of specific GF receptors in tumor cells [38].

The remarkably high levels of angiogenic regulators in activated platelets are results of not only their enhanced expression in platelets but also their uptake from plasma [39]. In addition to the angiogenic regulators, platelets sequester tumor-derived cytokines, which in turn instigate platelets to be recruited to responding tumor sites where they aid vessel formation [40]. 

Once activated, platelets form MPs, which are abundant in the circulation. The number of circulated platelet MPs increases in cancer patients compared to that in normal healthy controls [41,42]. The platelet MPs deliver angiogenic signals and can communicate with many types of cells to induce angiogenesis (Figure 1) [43]. An in vitro study on HUVECs demonstrates that platelet MPs facilitate capillary-like network formation in a dose-dependent manner. There is an upregulation of matrix metalloproteinases (MMPs) such as MMP-2 and MMP-9 in platelet MPs-stimulated HUVECs. The pro-angiogenic effects of platelet MPs which are reconfirmed in vivo in mice with a subcutaneous implantation of Matrigel are diminished totally in the loss of MMPs activity [44]. In addition to cancer, platelet MPs also activate angiogenesis in some other inflammatory diseases [45,46]. The angiogenetic signals delivered by platelet MPs consist of not only cytokines and GFs but also microRNAs to control gene expression as unraveled in recent studies. In the presence of platelet MPs, there is a substantial downregulation of anti-angiogenic modulators such as thrombospondin-1 (THBS-1) in HUVECs. The neovascularization effects of platelet MPs are explained by the transfer of miRNA let-7a, which directly targets the THBS-1 mRNA of HUVEC. The pro-angiogenic impact of platelets is depleted by treating the platelet MPs with RNAase [47]. Another platelet-originated miRNA with a crucial role in platelet angiogenic activity is miR-27b with autocrine effect. The transfection of miR-27b inhibits platelet THBS-1 synthesis, resulting in enhanced platelet-dependent endothelial tube formation in Matrigel [48]. On the other hand, miR-24, another miRNA which is transferred from platelet MPs to tumor cells, constrains tumor growth by targeting mitochondrial mt-Nd2, and Snora75, resulting in mitochondrial dysfunction and tumor suppression [49]. In addition to miR-24, platelets also produce several other anti-angiogenic factors. Interestingly, the pro-angiogenic factors and the anti-angiogenic factors are found to be located differently in platelets [50]. Furthermore, there are some dual-effect regulators derived from platelets, such as transforming growth factor-β (TGF-β). TGF-β regulates endothelial cells via two opposing type I receptor/Smad pathways. Activinreceptor-like kinase-1 (ALK1) enhances Smad1/5 phosphorylation, leading to endothelial cell activation, while ALK5 upregulates Smad2/3, suppressing endothelial cells [51]. An in vitro study with HUVECs shows that TGF-β modulates the binding between interleukin (IL)-37 and the ALK1 receptor complex, promoting endothelial cells proliferation and tube formation. TGFβ-mediated pro-angiogenesis is also supported in vivo with the mouse model of a Matrigel plug and oxygen-induced retinopathy (OIR) [52]. On the contrary, the addition of TGF-β1 to an in vitro model of vessel assembly in the presence of VEGF and FGF2 leads to diminished tube formation in a dose-dependent manner [53]. It seems that the overall TGF-β-related impact of platelets on angiogenesis is contextual and heterogeneous. 

Adjacent to major vasculogenic impacts on neoplastic epithelial cells and endothelial cells, activated platelets also facilitate angiogenesis through other cell types of the tumor-microenvironment. There is evidence that PDGF is essential for vascular pericytes and smooth muscle cells which help to stabilize the microvasculature. Blocking the PDGF receptor leads to pericyte and vascular smooth muscle degeneration, thereby promoting angiogenesis [54,55]. Recently, neuropeptide Y (NPY), a peptide that is involved in various physiological and homeostatic processes in both the central and the peripheral nervous systems, is also found in platelet lysate at a high level. NPY promotes migration and vessel formation of adipose tissue-originated stromal cells (ASCs). The loop number and network length found in Matrigel assays of ASCs co-cultured with platelet lysate are notably attenuated in parallel with decreased VEGF expression in the presence of a specific NPY receptor antagonist. Taken together, platelet NPY plays a vital role as a VEGF mediator in ASC angiogenesis [56]. Another type of cell-affecting endothelial homeostasis and function is fibroblasts. The supportive role of cancer-associated fibroblast (CAF) and CAF-derived galectin-1 in vessel formation is observed in gastric tumors [57]. Fibroblasts in general, and CAFs in particular, are regulated by platelet-derived growth factors such as basic fibroblast growth factor (bFGF) and PDGF. Therefore, platelets might have an indirect impact on tumor angiogenesis through fibroblasts. In fact, consecutive delivery of bFGF and PDGF leads to both the enhanced endothelial cell migration and the growth of vascular pericytes in vitro (Figure 1) [58].

### 3.2. Platelets and Tumor Metastasis 

Tumor metastasis consists of the detachment of tumor cells from the original tumor [59,60], entering the circulation, extravasation, and finally colonization at the new site, in all of which platelets play a significant role.

**Figure 2:**
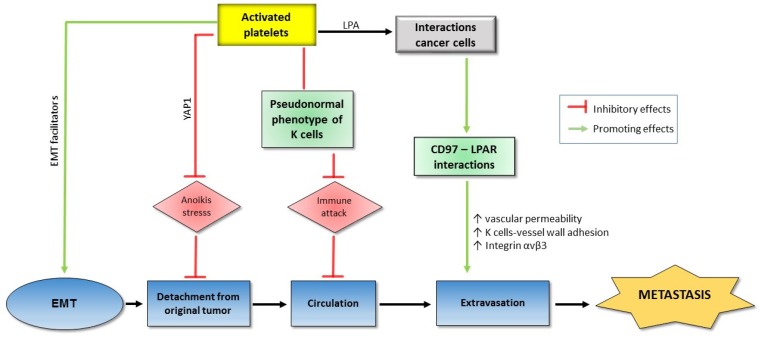
Platelets and tumor metastasis. Interactions between tumor cells and activated platelets, on the one hand, upregulate epithelial mesenchymal transition (EMT) facilitators and promote extravasation, and, on the other hand, protect the cancer cells from various dangers during the process of tumor detachment and circulation. As a result, tumor metastasis is promoted with supports of platelet ally (see text for details).

Firstly, before detaching from the tumor and intravasation, neoplastic epithelial cells undergo an epithelial mesenchymal transition (EMT). They become less adherent, less polar, more mesenchymal, and have increased mobility. The EMT is characterized by a decreased expression of E-cadherin following the increased expression of p38 and relating pathways (Figure 2) [61,62,63]. 

The role of platelets in EMT has been supported in various studies. Co-incubation with platelets potentiates a phenotypic change towards an EMT trend in ovarian cancer cells in conjunction with enhanced expression of tissue factor, a metastasis marker [64]. There is an activation of platelets by various factors produced from the cancer cells. The platelet activators expressed on tumor cell surfaces, on one hand, lead to the cancer cells-induced aggregation of platelets and, on the other hand, induce the EMT of tumor cells. It has been reported that in pancreatic cancer and stroma, there is a significant correlation between platelets activation levels (marked by CD42b expression) and EMT markers such as Snail 1 upregulation and E-cadherin downregulation [65]. Co-culturing HT29 colon carcinoma cells with platelets results in EMT of the cancer cells with decreased E-cadherin, increased Twist1, and enhanced cell mobility. All of these impacts are reversed by various antiplatelet agents such as aspirin, ticagrelor, and DG-041 (an antagonist of prostaglandin (PG)E_2_ EP3 receptor) [66].

The mechanism of the role of platelets in EMT has been revealed by many factors derived from either platelets or tumor to induce platelet activation. Podoplanin is a platelet activator secreted by cancer cells. Podoplanin-positive cancer cell lines promote platelet activation and especially platelet TGF-β expression which in turn triggers EMT transition of the cancer cells. Interestingly, TGF-β blockade significantly inhibits podoplanin-induced EMT and metastasis in mice injected with highly metastatic lung cancer cells, suggesting that podoplanin mediates tumor metastasis by mounting platelet-derived TGF-β [67]. Among the platelet-associated metastasis mediators, TGF-β plays an essential role. The pre-treatment of colon carcinoma cells or breast carcinoma cells promotes the TGF-β/Smad and NF-ĸB pathways, resulting in EMT phenotype, tumor migration, and invasion. Therefore, blocking NF-ĸB signaling or silencing platelet TGF-β leads to the inhibition of metastasis in mice injected with colon carcinoma cells [14]. The role of platelets in EMT of colon cancer cells is regulated by protease-activated receptor-1 (PAR1), a G protein-coupled receptor on the human platelet surface. The co-culture of colon cancer cells with PAR1-activated platelets promotes EMT with E-cadherin upregulation and vimentin downregulation in a dose-dependent manner. At the same time, PAR1-activated platelets inhibit expression of miR-200b, a TGF-β1-dependent EMT inhibitor in cancer cells [68]. Alternatively, acid sphingomyelinase (Asm) is an activated platelet-derived enzyme catalyzing the breakdown of sphingomyelin to ceramide and phosphorylcholine. Co-incubation of melanoma cells with either Asm or activated platelets but not with Asm-deficient platelets stimulates several intracellular signaling molecules such as p38 MAP kinase (p38K), phospholipase Cxγ (PLCxγ), ezrin, and extracellular signal-regulated kinases. As a result, the p38 pathway activates β1 integrin, an adherent factor which is crucial for tumor metastasis. The important role of the p38 pathway in metastasis is emphasized with the fact that the attenuation of p38K activity prevents both Asm-induced adherence and lung metastasis of melanoma cells [69]. Another relation of activated platelets to the p38 pathway is through the apoptosis signal-regulating kinase 1 (ASK1), an important kinase in both p38 and the c-Jun N-terminal kinase (JNK) pathways. It is shown that ASK1 builds up in activated platelets, leading to metastasis. ASK1 knockout mice show remarkably attenuated metastasis, downregulated p38 and JNK phosphorylation as well as coagulation disorders [70]. Furthermore, the contribution of platelets to metastasis also depends on autotaxin (ATX), through its regulation activity on levels of lysophosphatidic acid (LPA) [71]. LPA is an intermediate factor in the interaction between cancer cells and platelets for tumor invasion and metastasis. The signaling pathway through the LPA receptor is enhanced by CD97, a member of the epidermal growth factor which is activated in various types of cancer [72,73,74]. 

Anoikis is an apoptosis type that occurs when cells detach from the surrounding extracellular matrix (ECM). Anoikis becomes a barrier that cancer cells must overcome before metastasis. Recently, it is reported that co-incubation of multiple human cancer cell lines with platelets under artificial anoikis conditions activates and promotes the nuclear translocation of Yes-Associated Protein 1 (YAP1), a transcriptional regulator by activating genes involved in cell proliferation and suppressing apoptotic genes. Consequently, a number of anti-apoptotic genes are upregulated and anoikis is reversed. YAP1 silencing results in decreased metastasis induced by platelet transfusion in mice injected with human ovarian cancer cells (Figure 2) [75]. 

Alternatively, special local microenvironments (or niches) are crucial for metastasis. The metastasis niches which contain mostly ECM are formed with the contributions of platelets and then granulocytes. In a study on Lewis lung carcinoma spontaneous metastatic model, the knockout of platelet ADP receptor (P2Y12) leads to decreased lung fibronectin, a major component of ECM which is upregulated strongly in the connective tissue of a pre-metastatic organ, resulting in decreased pulmonary metastasis [76]. P2Y12 is the target for common anti-platelet drugs, so it may be developed into a new target for anti-metastatic drugs as well.

During circulation, before reaching the distant site, cancer cells are exposed to severe dangers such as shear forces, and immune attack. Platelets support cancer metastasis by protecting cancer cells from shear forces caused by blood flow. The co-incubation of A2780 ovarian cancer cells and human platelets notably lessens lactate dehydrogenase (a marker for shear-induced membrane damage) released by the cancer cells [77]. Another significant danger to metastasizing cells in the circulation is immune attack, in which natural killer (NK) cells play a key role in the immune system. On the one hand, TGF-β released from platelets attenuates the natural killer group 2D (NKG2D), an activating receptor on the NK surface [78]. On the other hand, in the blood, the interaction between metastasizing tumor cells and platelets results in co-expression of platelet-specific proteins, including the platelet-originated normal major histocompatibility complex (MHC) class I on the tumor cell surface. This kind of disguise diminishes cytotoxicity and interferon-γ secretion of NK cells [79]. Only a few cancer cells can manage to survive after circulation stresses, and the final border before colonization at the new site is extravasation which again depends on the support of platelets. In a study on Hodgkin lymphoma cell lines with the presence of platelets, there is a substantial increase in adherence between the cancer cells and the activated platelets, followed by the adherence of the complex to endothelial cells. The cell adhesions depend on CD15, P-selectin, and tumor necrosis factor-β (TNF-β) [80]. The role of platelets in extravasation is also supported in vivo. In mice injected with human tumor cells, the cancer cell–platelet interactions lead to the production of high-affinity integrin αvβ3 from cancer cells which facilitate vascular migration [81]. Recently, the role of CD97, an adhesion G protein-coupled receptor, which is overexpressed in a number of cancer types in tumor cell transendothelial migration, is supported both in vitro and in vivo. The induction of platelet activation in co-incubation with prostate cancer cells depends on the level of CD97. Moreover, supernatants from platelets co-cultured with cancer cells promote the dissociation of endothelial tight junctions while the impact is abolished in the same model using CD97-deficient cancer cells. Most importantly, the essential role of CD97 in tumor-induced mice is confirmed by the significant difference of vascular permeability and metastatic foci number between the wild type and the depleted phenotypes of CD97 (Figure 2) [82]. 

## 4. Platelets in Cancer Drug Resistance 

### 4.1. Chemotherapy

Various studies have indicated that the platelets from cancer patients who receive chemotherapy may influence the efficacy of drug treatment in many different cancer types [83]. For instance, high platelet levels are reported to attenuate the efficacy of platinum-based treatment in NSCLC [9]. Carboplatin and paclitaxel chemotherapy are also suggested to be a platelet-sparing drug combination [84]. It was observed that serum thrombopoietin levels in patients receiving carboplatin and paclitaxel were significantly enhanced five hours after infusion and remained elevated at day seven compared to those in normal controls [84]. Moreover, breast cancer patients with a low platelet-to-lymphocyte ratio, treated with neoadjuvant chemotherapy, can achieve a higher complete pathological response, independently of the primary tumor molecular subtype [85]. 

Additionally, the platelet-to-lymphocyte ratio and its dynamic changes during chemotherapy are useful to predict a more accurate prognosis of advanced biliary tract cancer [86]. For instance, after neoadjuvant chemotherapy, the platelet-to-lymphocyte ratio level could be negatively associated with survival prognosis in gastric cancer patients [87] and breast cancer patients [88]. Alternatively, the regeneration of hematopoiesis, especially the hyper-recovery of platelets after induction therapy, is considered as a significant predictor of relapse-free survival in patients with acute myeloid leukemia [89]. Taken together, as the sensitivity of megakaryocytic precursor cells to chemotherapeutics is suggested to vary, the extent of platelet dysfunction could be considered as a combined result of the sensitivity of the precise drugs administrated and their dosage [90,91] as summarized in Table 1.

### 4.2. Chemotherapy Resistance 

The treatment of cancer is often complicated by the lack of response to chemotherapy leading to chemo-resistance, which remains a major problem in anticancer drug therapy, and the association of platelets with chemo-resistance in cancer treatment has been also investigated. 

The principal mechanism regulating chemotherapy resistance is the elevated proliferation of cancer cells through the activation of anti-apoptotic proteins or phenotypic conversion in cancer cells through EMT, which could possibly be affected by platelets [92]. Platelets exhibit a pro-proliferative function through the secretion of various growth factors, and consequently possess the ability to counter the anti-proliferative effects of chemotherapeutic agents [92]. While the use of ABT-263 in patients is dose-limited due to causing thrombocytopenia via suppression of BCL-xL in platelets [93], loss of caspase-9 confers resistance to ABT-737, suppressing phosphatidylserine exposure and delaying APT-737-stimulated thrombocytopenia in vivo [94]. Additionally, the complex mechanism between gemcitabine/cisplatin and platelets lies in epithelialization. The releasing of TGF-β1 can (1) activate EMT in tumor tissues or (2) stimulate activated platelets through direct contact [14,66,95,96]. In pancreatic cancer cells, activated platelet-derived TGF-β1, rather than direct platelet–tumor cell contacts, promotes MAPK and PI3K/Akt signaling, resulting in the reduction of cisplatin sensitivity in these cells [97]. Consistently, emerging studies also indicate the critical contribution of platelets to chemotherapy resistance [92]. For instance, platelets can enhance adenocarcinoma cells survival and chemo-resistance to standard anticancer drugs [10]. Clinically, differential platelet levels can affect response to taxane-based therapy and platelet transfusion significantly counteracts the antitumor effect of chemotherapy in ovarian cancer [98]. Takeuchi et al. recently also observed the interaction of the novel platelet-enhancing agent eltrombopag with rosuvastatin via breast cancer resistance protein (BCRP) and suggested that BCRP in the small intestine can be the major target for interaction between rosuvastatin and eltrombopag in humans [99].

Alternatively, there are also several classical resistance mechanisms which might significantly contribute to drug resistance such as an increased number of drug efflux pumps located on the cell membrane or increased drug metabolism [100]. It has been suggested that platelets express relatively high amounts of multidrug resistance-associated protein 4 (MRP4), not only in the plasma membrane but also in the membrane of dense granules, eliciting relevance for modulator storage [101]. MRP4 confers resistance to nucleoside-based cytotoxic drugs [102], and the increase of MRP4, together with its specific localization during differentiation toward megakaryocytes, supports the concept of platelet-specific roles, whereas reduced transporter expression in leukocyte differentiation can have implications for chemotherapy [103]. Besides, the other efflux protein investigated in platelets is MRP1 and its physiological role is reported to be the active export of leukotriene C_4_, the highest-affinity substrate known for MRP1, from human platelets [101]. Platelet count and MRP1 are associated with a poor prognosis in various types of cancer [104,105]. Additionally, in hepatocellular carcinoma, the p0SK-Hep1 cells are resistant to both cisplatin- and doxorubicin-stimulated apoptosis, while cybrids (SK-Hep1Cyb) prepared by fusing p0SK-Hep1 cells with platelets display a restored susceptibility to both drugs, and this phenomenon is associated with P-glycoprotein and MRP1 [106]. 

Another mechanism by which platelets may also be associated with resistance to anti-angiogenic therapy is through the scavenging of anti-angiogenic agents. Both sunitinib and bevacizumab may be taken up by platelets, which might contribute to the bioavailability and pharmacodynamics of these anti-angiogenic agents [107]. Alternatively, during the infusions of G3139 (a Bcl-2 antisense oligodeoxynucleotide), there was a reduction of the absolute numbers of platelets and fatigue that precede vincristine, adriamycin, and dexamethasone (VAD) chemotherapy, suggesting that G3139 can overcome classical resistance and restore the sensitivity of myeloma tumor cells to VAD treatment [108]. Imatinib is highly effective in chronic-phase chronic myeloid leukemia (CML) patients resistant or intolerant to interferon, especially in those with normal platelet counts [109]. It has been also shown that platelet dysfunction is related to ponatinib, a new pan breakpoint cluster region-Abelson (BCR-ABL) inhibitor with efficacy for CML resistant to multiple tyrosine kinase inhibitor therapy [110]. 

The platelet activation index can also be suggested as a potential cancer biomarker for predicting chemotherapy resistance (Table 2). For example, a recent study reports that platelets may be useful cancer biomarkers for gemcitabine/cisplatin resistance in NSCLC treatment [111]. In castration-resistant patients, platelets can also harbor prostate cancer biomarkers and help to predict therapeutic response to abiraterone [112]. This may be because larger platelets can release more cytokines upon stimulation than smaller ones in cancer cells; subsequently, some cytokines can stimulate tumor promoters such as EMT or TGF-β, leading to chemotherapy resistance [111]. Additionally, there was a nonlinear association between naïve platelet counts and turnover resistance, regardless of aspirin regimen in myeloproliferative neoplasms [113]. Alternatively, IL-6 may also enhance chemotherapeutic resistance by suppressing the activation of proteases involved in apoptosis [114], and serum IL-6 levels are associated with poor survival in patients with hormone-refractory metastatic breast cancer [115].

## 5. Opportunities of Targeting Platelets in Cancer Treatment

### 5.1. Combinations of PDGFs/PDGFRs Inhibitors and Others

Although the PDGF isoforms and receptors’ function in resistance to tyrosine kinase inhibitors (TKIs) indicates a critical challenge to cancer treatment, combining different targeted agents may offer potential benefits for enhanced efficacy over monotherapy while maintaining a favorable safety compared with chemotherapy combinations [116]. Recent research developments focused on the suppression of only PDGFR without affecting EGFR [117]. However, it was investigated that EGFR may also undergo a heterodimerization with the associated PDGFR in various types of cancer similar to EGFR [118,119]. Additionally, it may also be possible to “sensitize” cells to antiangiogenic treatment by lowering the tumor cell survival threshold through suppressing EGFR signaling pathways [116]. Subsequently, a dual suppression of both PDGFR and EGFR may be preferable to prevent a kinase inhibitor resistance led by a receptor heterodimerization [120]. 

### 5.2. Platelets as Cancer Biomarkers 

The biomarkers are usually urgent and important for prognosis and personalized cancer medicine and the PDGFs/PDGFRs can also be critically considered as potential biomarkers, eliciting resistance to targeted therapies. For example, the involvement of PDGFs/PDGFRs activation in resistant TKIs may function as a prognostic biomarker and support the clinical development of PDGFs/PDGRs-targeted agents [121]. A recent study also investigated the role and presence of platelets in primary tumors and observed that the primary tumor cells related to platelets may exhibit chemo-resistance to common anticancer drugs such as taxanes and anthracycline [122]. Taken together, platelet aggregation can be an effective predictor of chemo-resistance and a novel therapeutic target for overcoming chemo-resistance, one of the major objects of cancer treatment. Additionally, platelets and their activation index can likely be used more extensively due to their low cost compared to serum tumor markers [111] and may address the problem of high costs of novel therapies by validating therapy responders [112].

## 6. Conclusions 

In spite of extensive efforts in cancer treatment improvement, drug resistance still remains a problematic challenge for both scientists and clinicians. In their strong association with tumors, activated platelets, especially their growth factors, potentiate chemo-resistance of cancer cells through regulation of tumor growth, angiogenesis, and metastasis. Therefore, targeting tumor–platelet interactions and more specifically targeting platelet-originated growth factors might be a promising approach in order to enhance tumor sensitivity to chemotherapy. Alternatively, and perhaps more feasibly, platelet index and platelet growth factors can be used as biomarkers to predict the response to a chemo-regimen in a personalized medicine approach, a critical trend in cancer treatment. These two platelet-based strategies should be the focus of more attention, in order to address the severe problem of cancer drug resistance.

## Figures and Tables

**Table 1:** The contributions of platelets to chemotherapy treatment in cancer cells.

Drug	Role of Platelets	Ref.
Platinum	High platelet levels attenuate the efficacy of platinum-based treatment in NSCLC.	[9]
Postoperative chemotherapy	The platelet-to-lymphocyte ratio is a potential marker of therapeutic effect of postoperative chemotherapy in non-metastatic esophageal squamous cell carcinoma.	[123]
Postoperative adjuvant chemotherapy	The platelet-to-lymphocyte ratio can be used to predict the prognosis of patients with NSCLC treated with postoperative adjuvant chemotherapy.	[124]
Chemotherapy	Platelets after chemotherapy enhance procoagulant activity and can affect the hypercoagulative state of NSCLC.	[125]
Resveratrol	Resveratrol inhibits pulmonary tumor metastasis by interrupting the platelet–tumor cell amplification loop.	[126]
Rituximab	Lack of detectable platelet autoantibodies is associated with nonresponsiveness to rituximab treatment in immune thrombocytopenia patients.	[127]
Chemotherapy	Pretreatment platelet-to-lymphocyte ratio is correlated with the response to first-line chemotherapy in patients with metastatic gastric cancer.	[128]
Neoadjuvant chemotherapy	Pretreatment platelet/lymphocyte can be a significant predictive indicator for neoadjuvant chemotherapy response in breast cancer patients.	[88]
Aspirin	Aspirin therapy decreases the ability of platelets to stimulate colorectal cancer cell proliferation.	[129]

**Table 2:** The contributions of platelets to the drug resistance of cancer cells.

Model/Cell Types	Corresponding Drugs	Role of Platelets	Ref
Pancreatic ductal adenocarcinoma (PDAC) cells	Gemcitabine	Platelets modulate cytidine deaminase (CDD) and human equilibrative nucleoside transporter 1, the markers expression of gemcitabine resistance in pancreatic cells. Platelet-derived ADP and ATP can active survival signals of cancer cells through modulating Slug and CDD levels.	[130]
Non-small cell lung cancer	Gemcitabine/Cisplatin	Platelet activation index can be a potential marker for predicting gemcitabine/cisplatin resistance	[111]
Castration cancer patients	Abiraterone	Platelets harbor prostate cancer biomarkers and the ability to predict therapeutic response.	[112]
Immune thrombocytopenia patients	Rituximab	Lack of detectable platelet autoantibodies is associated with non-responsiveness to rituximab treatment.	[127]
Non-small cell lung cancer	Crizotinib	Platelets are useful for predicting and monitoring outcome of crizotinib. Platelets are valuable biomarkers for the non-invasive detection of EML4-ALK rearrangements.	[131]
Serum in patients	Paclitaxel/Carboplatin	The platelet-sparing phenomenon could be observed in patients treated with carboplatin and paclitaxel chemotherapy.	[84]

## Abbreviations

ADP	adenosine diphosphate
Asm	acid sphingomyelinase
ATX	autotaxin
BFGF	basic fibroblast growth factor
CAF	cancer-associated fibroblast
CD	cluster differentiation
CDD	cytidine deaminase
CXCR4	chemokine receptor type 4
ECM	extracellular matrix
EGFR	epidermal growth factor receptor
EML4-ALK	echinoderm microtubule-associated protein-like 4-anaplastic lymphoma kinase
EMT	epithelial mesenchymal transition
EPCs	epithelial progenitor cells
fL	femtoliter
FLK-1	fetal liver kinase-1
GP	glycoprotein
hENT	human equilibrative transporter
HUVEC	human umbilical vein endothelial cell
JNK	c-Jun N-terminal kinases
LLC	Lewis lung carcinoma
LPA	lysophosphatidic acid
MHC	major histocompatibility complex
MMPs	matrix metalloproteinases
MPs	microparticles
MPV	mean platelet volume
NK	natural killer
NKG2D	natural killer group 2D
NPY	neuropeptide Y
P2Y12	platelet ADP receptor
PAR-1	protease-activated receptor-1
PDGF	platelet-derived growth factor
PDGFR	platelet-derived growth factor receptor
PDW	platelet distribution width
(PG)E_2_ EP3	prostaglandin E2 receptor subtype EP3
RTK	receptor tyrosine kinase
SDF-1	stromal cell-derived factor-1
TBHS-1	thrombospondin-1
TCIPA	tumor cell-induced platelet activation
TFs	tissue factors
TGF-β	transforming growth factor-β
TKI	tyrosine kinase inhibitor
TX	thromboxane
VEGF	vascular endothelial growth factors

## References

[1] Bach D.H., Hong J.Y., Park H.J., Lee S.K. (2017). The role of exosomes and miRNAs in drug-resistance of cancer cells. Int. J. Cancer.

[2] Bach D.H., Park H.J., Lee S.K. (2018). The Dual Role of Bone Morphogenetic Proteins in Cancer. Mol. Ther. Oncolytics.

[3] Bach D.H., Kim D., Bae S.Y., Kim W.K., Hong J.Y., Lee H.J., Rajasekaran N., Kwon S., Fan Y., Luu T.T. (2018). Targeting Nicotinamide N-Methyltransferase and miR-449a in EGFR-TKI-Resistant Non-Small-Cell Lung Cancer Cells. Mol. Ther. Nucleic Acids.

[4] Bach D.H., Luu T.T., Kim D., An Y.J., Park S., Park H.J., Lee S.K. (2018). BMP4 Upregulation Is Associated with Acquired Drug Resistance and Fatty Acid Metabolism in EGFR-Mutant Non-Small-Cell Lung Cancer Cells. Mol. Ther. Nucleic Acids.

[5] Bach D.H., Long N.P., Luu T.T., Anh N.H., Kwon S.W., Lee S.K. (2018). The Dominant Role of Forkhead Box Proteins in Cancer. Int. J. Mol. Sci..

[6] Bach D.H., Lee S.K. (2018). Long noncoding RNAs in cancer cells. Cancer Lett..

[7] Bach D.H., Lee S.K. (2018). The potential impacts of tylophora alkaloids and their derivatives in modulating inflammation, viral infections, and cancer. Curr. Med. Chem..

[8] Leary M., Heerboth S., Lapinska K., Sarkar S. (2018). Sensitization of Drug Resistant Cancer Cells: A Matter of Combination Therapy. Cancers.

[9] Wang Z., Fang M., Li J., Yang R., Du J., Luo Y. (2018). High Platelet Levels Attenuate the Efficacy of Platinum-Based Treatment in Non-Small Cell Lung Cancer. Cell Physiol. Biochem..

[10] Radziwon-Balicka A., Medina C., O’Driscoll L., Treumann A., Bazou D., Inkielewicz-Stepniak I., Radomski A., Jow H., Radomski M.W. (2012). Platelets increase survival of adenocarcinoma cells challenged with anticancer drugs: Mechanisms and implications for chemoresistance. Br. J. Pharmacol..

[11] Ankus E., Price S.J., Ukoumunne O.C., Hamilton W., Bailey S.E.R. (2018). Cancer incidence in patients with a high normal platelet count: A cohort study using primary care data. Fam. Pract..

[12] Bailey S.E.R., Ankus E., Price S., Pula G., Hamilton W. (2018). The diagnostic potential of high normal platelet counts for identifying cancer in primary care. Thromb. Res..

[13] Han H., Cao F.L., Wang B.Z., Mu X.R., Li G.Y., Wang X.W. (2014). Expression of angiogenesis regulatory proteins and epithelial-mesenchymal transition factors in platelets of the breast cancer patients. Sci. World J..

[14] Labelle M., Begum S., Hynes R.O. (2011). Direct signaling between platelets and cancer cells induces an epithelial-mesenchymal-like transition and promotes metastasis. Cancer Cell.

[15] Lomnytska M., Pinto R., Becker S., Engström U., Gustafsson S., Björklund C., Templin M., Bergstrand J., Xu L., Widengren J. (2018). Platelet protein biomarker panel for ovarian cancer diagnosis. Biomark. Res..

[16] Sabrkhany S., Kuijpers M.J.E., Knol J.C., Olde Damink S.W.M., Dingemans A.-M.C., Verheul H.M., Piersma S.R., Pham T.V., Griffioen A.W., oude Egbrink M.G.A. (2018). Exploration of the platelet proteome in patients with early-stage cancer. J. Proteom..

[17] Zhu X., Cao Y., Lu P., Kang Y., Lin Z., Hao T., Song Y. (2018). Evaluation of platelet indices as diagnostic biomarkers for colorectal cancer. Sci. Rep..

[18] Yin J.-B., Wang X., Zhang X., Liu L., Wang R.-T. (2018). Mean platelet volume predicts survival in pancreatic cancer patients with synchronous liver metastases. Sci. Rep..

[19] Chen S., Na N., Jian Z. (2017). Pretreatment platelet count as a prognostic factor in patients with pancreatic cancer: A systematic review and meta-analysis. Onco Targets Ther..

[20] Cui M.-M., Li N., Liu X., Yun Z.-Y., Niu Y., Zhang Y., Gao B., Liu T., Wang R.-T. (2017). Platelet distribution width correlates with prognosis of non-small cell lung cancer. Sci. Rep..

[21] Pan V., Siva P.N., Modery-Pawlowski C.L., Singh Sekhon U.D., Gupta A.S. (2015). Targeted killing of metastatic cells using a platelet-inspired drug delivery system. RSC Adv..

[22] Li J., Ai Y., Wang L., Bu P., Sharkey C.C., Wu Q., Wun B., Roy S., Shen X., King M.R. (2016). Targeted drug delivery to circulating tumor cells via platelet membrane-functionalized particles. Biomaterials.

[23] Wang M., Zhao J., Zhang L., Wei F., Lian Y., Wu Y., Gong Z., Zhang S., Zhou J., Cao K. (2017). Role of tumor microenvironment in tumorigenesis. J. Cancer.

[24] Bach D.H., Liu J.Y., Kim W.K., Hong J.Y., Park S.H., Kim D., Qin S.N., Luu T.T., Park H.J., Xu Y.N. (2017). Synthesis and biological activity of new phthalimides as potential anti-inflammatory agents. Bioorg. Med. Chem..

[25] Pipili-Synetos E., Papadimitriou E., Maragoudakis M.E. (1998). Evidence that platelets promote tube formation by endothelial cells on matrigel. Br. J. Pharmacol..

[26] Kisucka J., Butterfield C.E., Duda D.G., Eichenberger S.C., Saffaripour S., Ware J., Ruggeri Z.M., Jain R.K., Folkman J., Wagner D.D. (2006). Platelets and platelet adhesion support angiogenesis while preventing excessive hemorrhage. Proc. Natl. Acad. Sci. USA.

[27] Thomas G.M., Panicot-Dubois L., Lacroix R., Dignat-George F., Lombardo D., Dubois C. (2009). Cancer cell-derived microparticles bearing P-selectin glycoprotein ligand 1 accelerate thrombus formation in vivo. J. Exp. Med..

[28] Medina C., Harmon S., Inkielewicz I., Santos-Martinez M.J., Jones M., Cantwell P., Bazou D., Ledwidge M., Radomski M.W., Gilmer J.F. (2012). Differential inhibition of tumour cell-induced platelet aggregation by the nicotinate aspirin prodrug (ST0702) and aspirin. Br. J. Pharmacol..

[29] Lian L., Li W., Li Z.-Y., Mao Y.-X., Zhang Y.-T., Zhao Y.-M., Chen K., Duan W.-M., Tao M. (2013). Inhibition of MCF-7 breast cancer cell-induced platelet aggregation using a combination of antiplatelet drugs. Oncol. Lett..

[30] Caine G.J., Lip G.Y., Blann A.D. (2004). Platelet-derived VEGF, Flt-1, angiopoietin-1 and P-selectin in breast and prostate cancer: Further evidence for a role of platelets in tumour angiogenesis. Ann. Med..

[31] Chater C., Bauters A., Beugnet C., M’Ba L., Rogosnitzky M., Zerbib P. (2018). Intraplatelet Vascular Endothelial Growth Factor and Platelet-Derived Growth Factor: New Biomarkers in Carcinoembryonic Antigen-Negative Colorectal Cancer?. Gastrointest. Tumors.

[32] Qi C., Li B., Guo S., Wei B., Shao C., Li J., Yang Y., Zhang Q., Li J., He X. (2015). P-Selectin-Mediated Adhesion between Platelets and Tumor Cells Promotes Intestinal Tumorigenesis in Apc(Min/+) Mice. Int. J. Biol. Sci..

[33] Etulain J., Negrotto S., Tribulatti M.V., Croci D.O., Carabelli J., Campetella O., Rabinovich G.A., Schattner M. (2014). Control of angiogenesis by galectins involves the release of platelet-derived proangiogenic factors. PLoS ONE.

[34] Battinelli E.M., Markens B.A., Kulenthirarajan R.A., Machlus K.R., Flaumenhaft R., Italiano J.E. (2014). Anticoagulation inhibits tumor cell-mediated release of platelet angiogenic proteins and diminishes platelet angiogenic response. Blood.

[35] Li X., Hou J., Wu B., Chen T., Luo A. (2014). Effects of Platelet-rich Plasma and Cell Coculture on Angiogenesis in Human Dental Pulp Stem Cells and Endothelial Progenitor Cells. J. Endod..

[36] Jun H., Lei D., Qifang Y., Yuan X., Deqin Y. (2018). Effects of concentrated growth factors on the angiogenic properties of dental pulp cells and endothelial cells: An in vitro study. Braz. Oral Res..

[37] Dissanayaka W.L., Zhu L., Hargreaves K.M., Jin L., Zhang C. (2015). In vitro analysis of scaffold-free prevascularized microtissue spheroids containing human dental pulp cells and endothelial cells. J. Endod..

[38] Bilalis A., Pouliou E., Roussou M., Papanikolaou A., Tassidou A., Economopoulos T., Terpos E. (2017). Increased expression of platelet derived growth factor receptor beta on trephine biopsies correlates with advanced myeloma. J. BUON.

[39] Klement G.L., Yip T.T., Cassiola F., Kikuchi L., Cervi D., Podust V., Italiano J.E., Wheatley E., Abou-Slaybi A., Bender E. (2009). Platelets actively sequester angiogenesis regulators. Blood.

[40] Kuznetsov H.S., Marsh T., Markens B.A., Castaño Z., Greene-Colozzi A., Hay S.A., Brown V.E., Richardson A.L., Signoretti S., Battinelli E.M. (2012). Identification of luminal breast cancers that establish a tumor-supportive macroenvironment defined by proangiogenic platelets and bone marrow-derived cells. Cancer Discov..

[41] Nafady A., Nasreldin E., Nafady-Hego H., Nasif K., Abd-Elmawgoud E., Sayed M. (2018). Alteration of trace elements and T-cell subsets in patients with 946;-thalassemia major: Influence of high ferritin level. Egypt. J. Haematol..

[42] Tseng C.C., Wang C.C., Chang H.C., Tsai T.H., Chang L.T., Huang K.T., Leu S., Yen C.H., Liu S.F., Chen C.H. (2013). Levels of circulating microparticles in lung cancer patients and possible prognostic value. Dis. Mark..

[43] Varon D., Shai E. (2015). Platelets and their microparticles as key players in pathophysiological responses. J. Thromb. Haemost..

[44] Sun C., Feng S.B., Cao Z.W., Bei J.J., Chen Q., Zhao W.B., Xu X.J., Zhou Z., Yu Z.P., Hu H.Y. (2017). Up-Regulated Expression of Matrix Metalloproteinases in Endothelial Cells Mediates Platelet Microvesicle-Induced Angiogenesis. Cell Physiol. Biochem..

[45] Gaetani E., Del Zompo F., Marcantoni M., Gatto I., Giarretta I., Porfidia A., Scaldaferri F., Laterza L., Lopetuso L., Gasbarrini A. (2018). Microparticles Produced by Activated Platelets Carry a Potent and Functionally Active Angiogenic Signal in Subjects with Crohn’s Disease. Int. J. Mol. Sci..

[46] Suades R., Padro T., Alonso R., Mata P., Badimon L. (2015). High levels of TSP1+/CD142+ platelet-derived microparticles characterise young patients with high cardiovascular risk and subclinical atherosclerosis. Thromb. Haemost..

[47] Anene C., Graham A.M., Boyne J., Roberts W. (2018). Platelet microparticle delivered microRNA-Let-7a promotes the angiogenic switch. Biochim. Biophys. Acta Mol. Basis Dis..

[48] Miao X., Rahman M.F., Jiang L., Min Y., Tan S., Xie H., Lee L., Wang M., Malmstrom R.E., Lui W.O. (2018). Thrombin-reduced miR-27b attenuates platelet angiogenic activities in vitro via enhancing platelet synthesis of anti-angiogenic thrombospondin-1. J. Thromb. Haemost..

[49] Michael J.V., Wurtzel J.G.T., Mao G.F., Rao A.K., Kolpakov M.A., Sabri A., Hoffman N.E., Rajan S., Tomar D., Madesh M. (2017). Platelet microparticles infiltrating solid tumors transfer miRNAs that suppress tumor growth. Blood.

[50] Martin-Granado V., Ortiz-Rivero S., Carmona R., Gutierrez-Herrero S., Barrera M., San-Segundo L., Sequera C., Perdiguero P., Lozano F., Martin-Herrero F. (2017). C3G promotes a selective release of angiogenic factors from activated mouse platelets to regulate angiogenesis and tumor metastasis. Oncotarget.

[51] Mallet C., Vittet D., Feige J.J., Bailly S. (2006). TGFbeta1 induces vasculogenesis and inhibits angiogenic sprouting in an embryonic stem cell differentiation model: Respective contribution of ALK1 and ALK5. Stem Cells.

[52] Zhao M., Hu Y., Jin J., Yu Y., Zhang S., Cao J., Zhai Y., Wei R., Shou J., Cai W. (2017). Interleukin 37 promotes angiogenesis through TGF-β signaling. Sci. Rep..

[53] Ramsauer M., D’Amore P.A. (2007). Contextual role for angiopoietins and TGFβ1 in blood vessel stabilization. J. Cell Sci..

[54] Wilkinson-Berka J., Babic S., De Gooyer T., Stitt A., Jaworski K.G.T., Ong L., Kelly D., Gilbert R. (2004). Inhibition of Platelet-Derived Growth Factor Promotes Pericyte Loss and Angiogenesis in Ischemic Retinopathy. Am. J. Pathol..

[55] Ruan J., Luo M., Wang C., Fan L., Yang S.N., Cardenas M., Geng H., Leonard J.P., Melnick A., Cerchietti L. (2013). Imatinib disrupts lymphoma angiogenesis by targeting vascular pericytes. Blood.

[56] Businaro R., Scaccia E., Bordin A., Pagano F., Corsi M., Siciliano C., Capoano R., Procaccini E., Salvati B., Petrozza V. (2018). Platelet Lysate-Derived Neuropeptide y Influences Migration and Angiogenesis of Human Adipose Tissue-Derived Stromal Cells. Sci. Rep..

[57] Tang D., Gao J., Wang S., Ye N., Chong Y., Huang Y., Wang J., Li B., Yin W., Wang D. (2016). Cancer-associated fibroblasts promote angiogenesis in gastric cancer through galectin-1 expression. Tumour Biol..

[58] Tengood J.E., Ridenour R., Brodsky R., Russell A.J., Little S.R. (2011). Sequential delivery of basic fibroblast growth factor and platelet-derived growth factor for angiogenesis. Tissue Eng. Part A.

[59] Kim W.K., Bach D.H., Ryu H.W., Oh J., Park H.J., Hong J.Y., Song H.H., Eum S., Bach T.T., Lee S.K. (2017). Cytotoxic activities of Telectadium dongnaiense and its constituents by inhibition of the Wnt/beta-catenin signaling pathway. Phytomedicine.

[60] van Dalum G., Holland L., Terstappen L.W. (2012). Metastasis and Circulating Tumor Cells. EJIFCC.

[61] Cheng J.C., Klausen C., Leung P.C. (2010). Hydrogen peroxide mediates EGF-induced down-regulation of E-cadherin expression via p38 MAPK and snail in human ovarian cancer cells. Mol. Endocrinol..

[62] Ling G., Ji Q., Ye W., Ma D., Wang Y. (2016). Epithelial-mesenchymal transition regulated by p38/MAPK signaling pathways participates in vasculogenic mimicry formation in SHG44 cells transfected with TGF-β cDNA loaded lentivirus in vitro and in vivo. Int. J. Oncol..

[63] Wen S., Hou Y., Fu L., Xi L., Yang D., Zhao M., Qin Y., Sun K., Teng Y., Liu M. (2019). Cancer-associated fibroblast (CAF)-derived IL32 promotes breast cancer cell invasion and metastasis via integrin β3–p38 MAPK signalling. Cancer Lett..

[64] Orellana R., Kato S., Erices R., Bravo M.L., Gonzalez P., Oliva B., Cubillos S., Valdivia A., Ibanez C., Branes J. (2015). Platelets enhance tissue factor protein and metastasis initiating cell markers, and act as chemoattractants increasing the migration of ovarian cancer cells. BMC Cancer.

[65] Miyashita T., Tajima H., Makino I., Nakagawara H.H.N., Kitagawa H., Duncan M.D., Harmon J.W., Ohta T. (2014). The metastasis-promoting roles of extravasated platelet aggregation in pancreatic cancer and stroma. J. Am. Coll. Surg..

[66] Guillem-Llobat P., Dovizio M., Bruno A., Ricciotti E., Cufino V., Sacco A., Grande R., Alberti S., Arena V., Cirillo M. (2016). Aspirin prevents colorectal cancer metastasis in mice by splitting the crosstalk between platelets and tumor cells. Oncotarget.

[67] Takemoto A., Okitaka M., Takagi S., Takami M., Sato S., Nishio M., Okumura S., Fujita N. (2017). A critical role of platelet TGF-β release in podoplanin-mediated tumour invasion and metastasis. Sci. Rep..

[68] Jia Y., Zhang S., Miao L., Wang J., Jin Z., Gu B., Duan Z., Zhao Z., Ma S., Zhang W. (2016). [Corrigendum] Activation of platelet protease-activated receptor-1 induces epithelial-mesenchymal transition and chemotaxis of colon cancer cell line SW620. Oncol. Rep..

[69] Carpinteiro A., Beckmann N., Seitz A., Hessler G., Wilker B., Soddemann M., Helfrich I., Edelmann B., Gulbins E., Becker K.A. (2016). Role of Acid Sphingomyelinase-Induced Signaling in Melanoma Cells for Hematogenous Tumor Metastasis. Cell Physiol. Biochem..

[70] Kamiyama M., Naguro I., Ichijo H. (2016). PO-16 - ASK1 regulates tumor lung metastasis and platelet functions. Thromb. Res..

[71] Leblanc R., Lee S.C., David M., Bordet J.C., Norman D.D., Patil R., Miller D., Sahay D., Ribeiro J., Clezardin P. (2014). Interaction of platelet-derived autotaxin with tumor integrin alphaVbeta3 controls metastasis of breast cancer cells to bone. Blood.

[72] Ward Y., Lake R., Martin P.L., Killian K., Salerno P., Wang T., Meltzer P., Merino M., Cheng S.Y., Santoro M. (2013). CD97 amplifies LPA receptor signaling and promotes thyroid cancer progression in a mouse model. Oncogene.

[73] Safaee M., Fakurnejad S., Bloch O., Clark A.J., Ivan M.E., Sun M.Z., Oh T., Phillips J.J., Parsa A.T. (2015). Proportional upregulation of CD97 isoforms in glioblastoma and glioblastoma-derived brain tumor initiating cells. PLoS ONE.

[74] Yin Y., Xu X., Tang J., Zhang W., Zhangyuan G., Ji J., Deng L., Lu S., Zhuo H., Sun B. (2018). CD97 Promotes Tumor Aggressiveness Through the Traditional G Protein-Coupled Receptor-Mediated Signaling in Hepatocellular Carcinoma. Hepatology.

[75] Haemmerle M., Taylor M.L., Gutschner T., Pradeep S., Cho M.S., Sheng J., Lyons Y.M., Nagaraja A.S., Dood R.L., Wen Y. (2017). Platelets reduce anoikis and promote metastasis by activating YAP1 signaling. Nat. Commun..

[76] Wang Y., Sun Y., Li D., Zhang L., Wang K., Zuo Y., Gartner T.K., Liu J. (2013). Platelet P2Y12 is involved in murine pulmonary metastasis. PLoS ONE.

[77] Egan K., Cooke N., Kenny D. (2014). Living in shear: Platelets protect cancer cells from shear induced damage. Metab. Clin. Exp..

[78] Kopp H.G., Placke T., Salih H.R. (2009). Platelet-derived transforming growth factor-beta down-regulates NKG2D thereby inhibiting natural killer cell antitumor reactivity. Cancer Res..

[79] Placke T., Orgel M., Schaller M., Jung G., Rammensee H.G., Kopp H.G., Salih H.R. (2012). Platelet-derived MHC class I confers a pseudonormal phenotype to cancer cells that subverts the antitumor reactivity of natural killer immune cells. Cancer Res..

[80] Ohana O.M., Ozer J., Prinsloo I., Benharroch D., Gopas J. (2015). Hodgkin lymphoma cell lines bind to platelets. Incubation with platelets induces CD15 and P-selectin dependent adhesion of the cell lines to Human Umbilical Vein Endothelial cells (HUVEC). Cancer Biol. Ther..

[81] Weber M.R., Zuka M., Lorger M., Tschan M., Torbett B.E., Zijlstra A., Quigley J.P., Staflin K., Eliceiri B.P., Krueger J.S. (2016). Activated tumor cell integrin alphavbeta3 cooperates with platelets to promote extravasation and metastasis from the blood stream. Thromb. Res..

[82] Ward Y., Lake R., Faraji F., Sperger J., Martin P., Gilliard C., Ku K.P., Rodems T., Niles D., Tillman H. (2018). Platelets Promote Metastasis via Binding Tumor CD97 Leading to Bidirectional Signaling that Coordinates Transendothelial Migration. Cell Rep..

[83] Haemmerle M., Stone R.L., Menter D.G., Afshar-Kharghan V., Sood A.K. (2018). The Platelet Lifeline to Cancer: Challenges and Opportunities. Cancer Cell.

[84] Pertusini E., Ratajczak J., Majka M., Vaughn D., Ratajczak M.Z., Gewirtz A.M. (2001). Investigating the platelet-sparing mechanism of paclitaxel/carboplatin combination chemotherapy. Blood.

[85] Cuello-Lopez J., Fidalgo-Zapata A., Lopez-Agudelo L., Vasquez-Trespalacios E. (2018). Platelet-to-lymphocyte ratio as a predictive factor of complete pathologic response to neoadjuvant chemotherapy in breast cancer. PLoS ONE.

[86] Cho K.M., Park H., Oh D.Y., Kim T.Y., Lee K.H., Han S.W., Im S.A., Kim T.Y., Bang Y.J. (2017). Neutrophil-to-lymphocyte ratio, platelet-to-lymphocyte ratio, and their dynamic changes during chemotherapy is useful to predict a more accurate prognosis of advanced biliary tract cancer. Oncotarget.

[87] Gong W., Zhao L., Dong Z., Dou Y., Liu Y., Ma C., Qu X. (2018). After neoadjuvant chemotherapy platelet/lymphocyte ratios negatively correlate with prognosis in gastric cancer patients. J. Clin. Lab. Anal..

[88] Xu J., Ni C., Ma C., Zhang L., Jing X., Li C., Liu Y., Qu X. (2017). Association of neutrophil/lymphocyte ratio and platelet/lymphocyte ratio with ER and PR in breast cancer patients and their changes after neoadjuvant chemotherapy. Clin. Transl. Oncol..

[89] Yamazaki E., Kanamori H., Itabashi M., Ogusa E., Numata A., Yamamoto W., Ito S., Tachibana T., Hagihara M., Matsumoto K. (2017). Hyper-recovery of platelets after induction therapy is a predictor of relapse-free survival in acute myeloid leukemia. Leuk. Lymphoma.

[90] Baaten C., Moenen F., Henskens Y.M.C., Swieringa F., Wetzels R.J.H., van Oerle R., Heijnen H.F.G., Ten Cate H., Holloway G.P., Beckers E.A.M. (2018). Impaired mitochondrial activity explains platelet dysfunction in thrombocytopenic cancer patients undergoing chemotherapy. Haematologica.

[91] Zeuner A., Signore M., Martinetti D., Bartucci M., Peschle C., De Maria R. (2007). Chemotherapy-induced thrombocytopenia derives from the selective death of megakaryocyte progenitors and can be rescued by stem cell factor. Cancer Res..

[92] Elaskalani O., Berndt M.C., Falasca M., Metharom P. (2017). Targeting Platelets for the Treatment of Cancer. Cancers.

[93] Polier G., Giaisi M., Kohler R., Muller W.W., Lutz C., Buss E.C., Krammer P.H., Li-Weber M. (2015). Targeting CDK9 by wogonin and related natural flavones potentiates the anti-cancer efficacy of the Bcl-2 family inhibitor ABT-263. Int. J. Cancer.

[94] White M.J., Schoenwaelder S.M., Josefsson E.C., Jarman K.E., Henley K.J., James C., Debrincat M.A., Jackson S.P., Huang D.C., Kile B.T. (2012). Caspase-9 mediates the apoptotic death of megakaryocytes and platelets, but is dispensable for their generation and function. Blood.

[95] Sharma D., Brummel-Ziedins K.E., Bouchard B.A., Holmes C.E. (2014). Platelets in tumor progression: A host factor that offers multiple potential targets in the treatment of cancer. J. Cell Physiol..

[96] Naugler W.E., Karin M. (2008). The wolf in sheep’s clothing: The role of interleukin-6 in immunity, inflammation and cancer. Trends Mol. Med..

[97] Chen H., Lan X., Liu M., Zhou B., Wang B., Chen P. (2013). Direct TGF-beta1 signaling between activated platelets and pancreatic cancer cells primes cisplatin insensitivity. Cell Biol. Int..

[98] Bottsford-Miller J., Choi H.J., Dalton H.J., Stone R.L., Cho M.S., Haemmerle M., Nick A.M., Pradeep S., Zand B., Previs R.A. (2015). Differential platelet levels affect response to taxane-based therapy in ovarian cancer. Clin. Cancer Res..

[99] Takeuchi K., Sugiura T., Matsubara K., Sato R., Shimizu T., Masuo Y., Horikawa M., Nakamichi N., Ishiwata N., Kato Y. (2014). Interaction of novel platelet-increasing agent eltrombopag with rosuvastatin via breast cancer resistance protein in humans. Drug Metab. Dispos..

[100] Broxterman H.J., Lankelma J., Hoekman K. (2003). Resistance to cytotoxic and anti-angiogenic anticancer agents: Similarities and differences. Drug Resist. Updat..

[101] Kock K., Grube M., Jedlitschky G., Oevermann L., Siegmund W., Ritter C.A., Kroemer H.K. (2007). Expression of adenosine triphosphate-binding cassette (ABC) drug transporters in peripheral blood cells: Relevance for physiology and pharmacotherapy. Clin. Pharmacokinet..

[102] Ritter C.A., Jedlitschky G., Meyer zu Schwabedissen H., Grube M., Kock K., Kroemer H.K. (2005). Cellular export of drugs and signaling molecules by the ATP-binding cassette transporters MRP4 (ABCC4) and MRP5 (ABCC5). Drug Metab. Rev..

[103] Oevermann L., Scheitz J., Starke K., Kock K., Kiefer T., Dolken G., Niessen J., Greinacher A., Siegmund W., Zygmunt M. (2009). Hematopoietic stem cell differentiation affects expression and function of MRP4 (ABCC4), a transport protein for signaling molecules and drugs. Int. J. Cancer.

[104] Fang L., Sheng H., Wan D., Zhu C., Jiang R., Sun X., Feng J. (2018). Prognostic role of multidrug resistance-associated protein 1 expression and platelet count in operable non-small cell lung cancer. Oncol. Lett..

[105] Jedlitschky G., Greinacher A., Kroemer H.K. (2012). Transporters in human platelets: Physiologic function and impact for pharmacotherapy. Blood.

[106] Ling X., He Y., Zhang G., Zhou Y., Yan B. (2012). Increased P-glycoprotein expression in mitochondria is related to acquired multidrug resistance in human hepatoma cells depleted of mitochondrial DNA. Int. J. Oncol..

[107] Huijbers E.J., van Beijnum J.R., Thijssen V.L., Sabrkhany S., Nowak-Sliwinska P., Griffioen A.W. (2016). Role of the tumor stroma in resistance to anti-angiogenic therapy. Drug Resist. Updat..

[108] van de Donk N.W., de Weerdt O., Veth G., Eurelings M., van Stralen E., Frankel S.R., Hagenbeek A., Bloem A.C., Lokhorst H.M. (2004). G3139, a Bcl-2 antisense oligodeoxynucleotide, induces clinical responses in VAD refractory myeloma. Leukemia.

[109] Cervantes F., Hernandez-Boluda J.C., Steegmann J.L., Conde E., Alvarez-Larran A., Lopez-Jimenez J., Osorio S., Villalon L., Camos M., Garcia-Conde J. (2003). Imatinib mesylate therapy of chronic phase chronic myeloid leukemia resistant or intolerant to interferon: Results and prognostic factors for response and progression-free survival in 150 patients. Haematologica.

[110] Neelakantan P., Marin D., Laffan M., Goldman J., Apperley J., Milojkovic D. (2012). Platelet dysfunction associated with ponatinib, a new pan BCR-ABL inhibitor with efficacy for chronic myeloid leukemia resistant to multiple tyrosine kinase inhibitor therapy. Haematologica.

[111] Shi L., Li Y., Yu T., Wang Z., Zhou C., Xing W., Xu G., Tong B., Zheng Y., Zhou J. (2018). Predictable Resistance and Overall Survival of Gemcitabine/Cisplatin by Platelet Activation Index in Non-Small Cell Lung Cancer. Med. Sci. Monit..

[112] Tjon-Kon-Fat L.A., Lundholm M., Schroder M., Wurdinger T., Thellenberg-Karlsson C., Widmark A., Wikstrom P., Nilsson R.J.A. (2018). Platelets harbor prostate cancer biomarkers and the ability to predict therapeutic response to abiraterone in castration resistant patients. Prostate.

[113] Perrier-Cornet A., Ianotto J.C., Mingant F., Perrot M., Lippert E., Galinat H. (2018). Decreased turnover aspirin resistance by bidaily aspirin intake and efficient cytoreduction in myeloproliferative neoplasms. Platelets.

[114] Badache A., Hynes N.E. (2001). Interleukin 6 inhibits proliferation and, in cooperation with an epidermal growth factor receptor autocrine loop, increases migration of T47D breast cancer cells. Cancer Res..

[115] Bachelot T., Ray-Coquard I., Menetrier-Caux C., Rastkha M., Duc A., Blay J.Y. (2003). Prognostic value of serum levels of interleukin 6 and of serum and plasma levels of vascular endothelial growth factor in hormone-refractory metastatic breast cancer patients. Br. J. Cancer.

[116] Tabernero J. (2007). The role of VEGF and EGFR inhibition: Implications for combining anti-VEGF and anti-EGFR agents. Mol. Cancer Res..

[117] Zhang L., Zheng Q., Yang Y., Zhou H., Gong X., Zhao S., Fan C. (2014). Synthesis and in vivo SAR study of indolin-2-one-based multi-targeted inhibitors as potential anticancer agents. Eur. J. Med. Chem..

[118] Saito Y., Haendeler J., Hojo Y., Yamamoto K., Berk B.C. (2001). Receptor heterodimerization: Essential mechanism for platelet-derived growth factor-induced epidermal growth factor receptor transactivation. Mol. Cell. Biol..

[119] Black P.C., Brown G.A., Dinney C.P., Kassouf W., Inamoto T., Arora A., Gallagher D., Munsell M.F., Bar-Eli M., McConkey D.J. (2011). Receptor heterodimerization: A new mechanism for platelet-derived growth factor induced resistance to anti-epidermal growth factor receptor therapy for bladder cancer. J. Urol..

[120] Fischer T., Najjar K., Totzke F., Schachtele C., Sippl W., Ritter C., Hilgeroth A. (2018). Discovery of novel dual inhibitors of receptor tyrosine kinases EGFR and PDGFR-beta related to anticancer drug resistance. J. Enzyme Inhib. Med. Chem..

[121] Wang Y., Appiah-Kubi K., Wu M., Yao X., Qian H., Wu Y., Chen Y. (2016). The platelet-derived growth factors (PDGFs) and their receptors (PDGFRs) are major players in oncogenesis, drug resistance, and attractive oncologic targets in cancer. Growth Factors.

[122] Ishikawa S., Miyashita T., Inokuchi M., Hayashi H., Oyama K., Tajima H., Takamura H., Ninomiya I., Ahmed A.K., Harman J.W. (2016). Platelets surrounding primary tumor cells are related to chemoresistance. Oncol. Rep..

[123] Yang Y., Xu H., Zhou L., Deng T., Ning T., Liu R., Zhang L., Wang X., Ge S., Li H. (2018). Platelet to lymphocyte ratio is a predictive marker of prognosis and therapeutic effect of postoperative chemotherapy in non-metastatic esophageal squamous cell carcinoma. Clin. Chim. Acta.

[124] Toda M., Tsukioka T., Izumi N., Komatsu H., Okada S., Hara K., Miyamoto H., Ito R., Shibata T., Nishiyama N. (2018). Platelet-to-lymphocyte ratio predicts the prognosis of patients with non-small cell lung cancer treated with surgery and postoperative adjuvant chemotherapy. Thorac. Cancer.

[125] Ma R., Bi Y., Kou J., Zhou J., Shi J. (2017). Enhanced procoagulant activity of platelets after chemotherapy in non-small cell lung cancer. Cancer Biol. Ther..

[126] He L., Fan F., Hou X., Gao C., Meng L., Meng S., Huang S., Wu H. (2017). Resveratrol suppresses pulmonary tumor metastasis by inhibiting platelet-mediated angiogenic responses. J. Surg. Res..

[127] Porcelijn L., Huiskes E., Schipperus M., van der Holt B., de Haas M., Zwaginga J.J. (2017). Lack of detectable platelet autoantibodies is correlated with nonresponsiveness to rituximab treatment in ITP patients. Blood.

[128] Wang J., Qu J., Li Z., Che X., Liu J., Teng Y., Jin B., Zhao M., Liu Y., Qu X. (2018). Pretreatment platelet-to-lymphocyte ratio is associated with the response to first-line chemotherapy and survival in patients with metastatic gastric cancer. J. Clin. Lab. Anal..

[129] Mitrugno A., Sylman J.L., Ngo A.T., Pang J., Sears R.C., Williams C.D., McCarty O.J. (2017). Aspirin therapy reduces the ability of platelets to promote colon and pancreatic cancer cell proliferation: Implications for the oncoprotein c-MYC. Am. J. Physiol. Cell Physiol..

[130] Elaskalani O., Falasca M., Moran N., Berndt M.C., Metharom P. (2017). The Role of Platelet-Derived ADP and ATP in Promoting Pancreatic Cancer Cell Survival and Gemcitabine Resistance. Cancers.

[131] Nilsson R.J., Karachaliou N., Berenguer J., Gimenez-Capitan A., Schellen P., Teixido C., Tannous J., Kuiper J.L., Drees E., Grabowska M. (2016). Rearranged EML4-ALK fusion transcripts sequester in circulating blood platelets and enable blood-based crizotinib response monitoring in non-small-cell lung cancer. Oncotarget.

